# Conducting a VR Clinical Trial in the Era of COVID-19

**DOI:** 10.3389/frvir.2021.639478

**Published:** 2021-04-29

**Authors:** Joy Stradford, Ashwin Sakhare, Roshan Ravichandran, E. Todd Schroeder, Lori A. Michener, Judy Pa

**Affiliations:** 1 Department of Neurology, Mark and Mary Stevens Neuroimaging and Informatics Institute, University of Southern California, Los Angeles, CA, United States,; 2 Department of Biomedical Engineering, Viterbi School of Engineering, University of Southern California, Los Angeles, CA, United States,; 3 Division of Biokinesiology and Physical Therapy, University of Southern California, Los Angeles, CA, United States,; 4 University of Southern California Alzheimer’s Disease Research Center, Department of Neurology, Keck School of Medicine of the University of Southern California, Los Angeles, CA, United States

**Keywords:** virtual reality, video games, aging, COVID-19, brain health, physical activity

## Abstract

The outbreak of severe acute respiratory syndrome coronavirus 2, also known as Coronavirus Disease 2019 (COVID-19) sparked a global public health pandemic that has impacted every aspect of daily life. Medical research was affected, and many clinical trials were halted to minimize COVID-19 transmission risk and spread while the world navigated this novel virus. Here we describe the relaunch of our virtual reality (VR) pilot clinical trial that uses an in-lab brain and body training program to promote brain health in mid-to-late life older adults, in the era of COVID-19. This case series includes five healthy female participants between 51 and 76 years of age, a subset of a larger VR pilot clinical trial that started pre-pandemic. We developed a revised study protocol based on the Center for Disease Control and World Health Organization guidelines to help manage the spread of COVID-19. Since the limited resumption of clinical trials at our institution in August 2020, we successfully completed over 200 in-lab virtual reality training sessions using our revised protocol. During this time, none of the five participants or three study staff reported any COVID-19 symptoms or reported a positive COVID-19 test. More than 40 voluntary COVID-19 tests were completed by our study staff over the last 6 months. All participants rated our safety protocol as very satisfied or extremely satisfied and that they would be very likely or extremely likely to participate in a VR clinical trial during the pandemic. Based on these findings, we suggest that continued VR clinical trial research during the COVID-19 pandemic is achievable and can be safely resumed if specific safety protocols are in place to mitigate the risk of exposure and spread of COVID-19.

## INTRODUCTION

Severe acute respiratory syndrome coronavirus 2, also known as Coronavirus Disease 2019 (COVID-19), is an infectious respiratory illness believed to spread primarily by person-to-person contact (Chu et al., 2020). Inhaling aerosols or respiratory droplets from an infected individual who coughs, sneezes, or speaks contributes to disease spread (Kutter et al., 2018). Droplets by definition are larger than 5 μm and usually fall directly to the ground or on to surfaces after human secretion (Atkinson et al., 2009). Aerosols are smaller than 5 μm and have a low settling velocity which causes them to remain in the air for longer periods of time (Ge et al., 2020). This low settling velocity allows the disease to become airborne and increases the risk of transmission. Coronavirus disease is marked by upper respiratory and gastrointestinal symptoms accompanied by a fever (Dhama et al., 2020). Other symptoms include loss of taste and/or smell, headache, muscle soreness, and fatigue. Older adults (55+) are at an increased risk of becoming critically ill or dying from contracting this virus, because viral infections progress more rapidly in weaker immune systems (Rothan and Byrareddy, 2020). Others at high risk include those with type 2 diabetes, obesity, heart disease, cancer, HIV, cardiovascular problems, or autoimmune disorders (Mayo Clinic, 2020). Minorities and other marginalized groups have been disproportionately affected by this virus (Laurencin and McClinton, 2020).

In addition to this global health crisis that has impacted many aspects of daily life (Ayittey et al., 2020; Stawicki et al., 2020), the pandemic has forced an unprecedented halt on research and clinical trials due to concerns of COVID-19 exposure and disease spreading (Sathian et al., 2020). Clinical trials research is a critical pillar of treatment discovery for chronic illnesses and informs clinical decisions (Umscheid et al., 2011). Virtual reality (VR) interventions serve specific patient populations to positively affect health outcomes. VR interventions are used to treat and offset conditions like generalized anxiety disorders (Repetto et al., 2013), obsessive-compulsive disorder (Ferreri et al., 2019), panic disorder (Pompoli et al., 2016), acute, episodic, and chronic stress (Rizzo et al., 2012), PTSD (Park et al., 2019), and memory disorders (Montana et al., 2019). VR immersion, stimulation, and exposure therapy have been used to combat severe phobias (Maples-Keller et al., 2017), in addition to stroke rehabilitation and motor-cognitive interventions (Kim et al., 2019; Juliano and Liew, 2020; Marin-Pardo et al., 2020). The resumption of VR clinical trials can have a direct clinically relevant impact on health outcomes. Therefore, now with more knowledge of ways to mitigate COVID-19 transmission risk, it is imperative that protocols with safeguards in place are developed and used to protect research participants and study staff while participating in clinical trials.

This case series provides methods for launching a clinical trial in the era of COVID-19. We describe the safety protocol and outcomes from our VR pilot clinical trial that uses an in-lab brain and body training program to promote brain health in mid-to-late life older adults.

## METHODS

This case series included five older adult participants (age: 51–76 years, five women) who were recruited to participate in a larger VR pilot clinical trial (Clinicaltrials.gov registration #: NCT04678778). Participants were recruited by campus flyers and participant referral. Inclusion criteria included those who were 50–85 years of age, physically able to ride a bike, fluent in English, willing to undergo magnetic resonance imaging (MRI) scans and blood draws, and willing and able to come to campus three times a week for a 12-week period. Exclusion criteria included major traumatic brain injuries, people with claustrophobia or ferromagnetic metal implants, and those with cancer who have undergone chemotherapy. Participants were given monetary compensation in the amount of $120 total distributed evenly across two time points, baseline and follow-up. This remuneration could potentially play a role in their willingness to participate in future studies.

Due to the onset of the COVID-19 pandemic, our study was interrupted on March 18, 2020 and research was halted for 5 months. We followed university guidelines to reopen and resume research on August 17, 2020. Briefly, participants underwent a cognitive and physical activity intervention by cycling on a stationary bike while wearing a VR head mounted display (HMD) in an immersive VR environment. The VR cycling intervention was ~60 min per session, three sessions a week, for 12 weeks. Due to safety concerns regarding COVID-19 infection or transmission risk, our study protocol followed the Center for Disease Control (CDC) and World Health Organization (WHO) COVID-19 recommendations as shown in Tables 1, 2, respectively, and described in further detail below. We were able to meet these CDC recommendations based on our financial support as an NIH-funded study.

### VR Room Setup and Configuration

#### Droplet and Aerosol Disease Transmission

**FIGURE 1 |:**
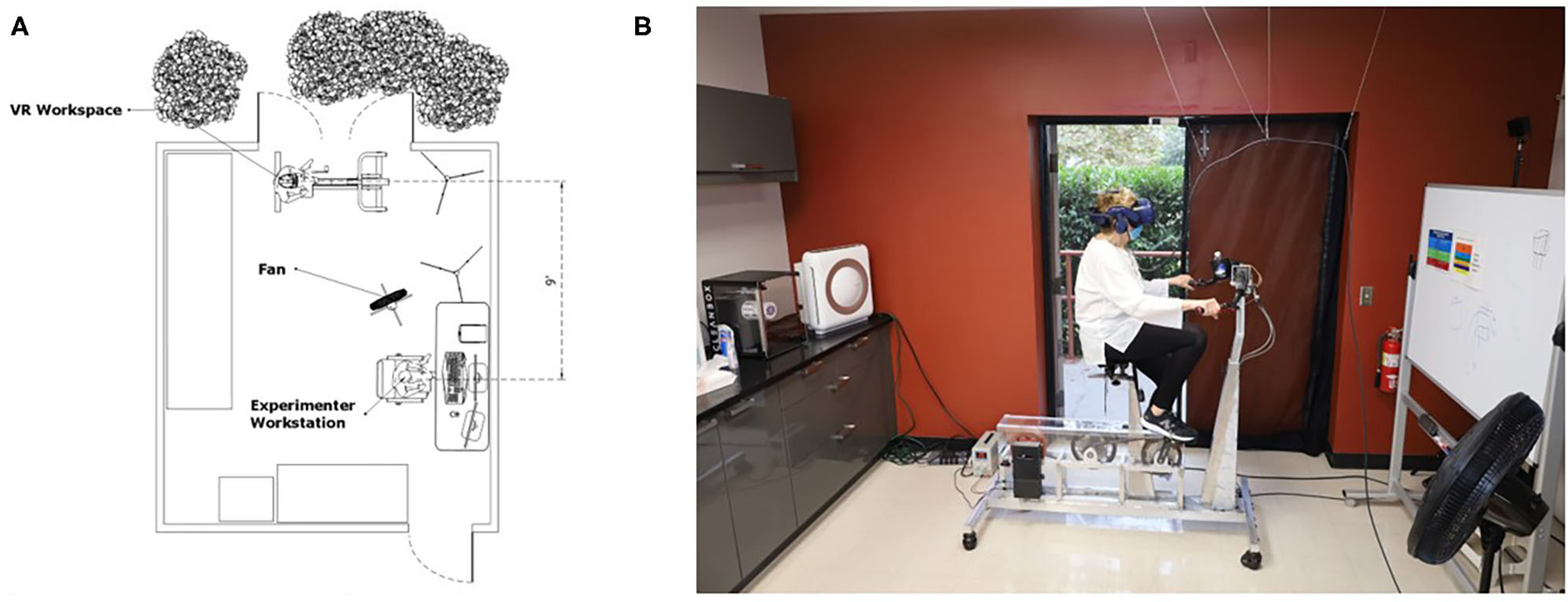
Room setup. Sketch of a top-down view of the room setup and configuration depicting CDC recommended 6 ft distance between researcher and participant, and open doors and fan for increased air flow and ventilation **(A)**. Real-world photograph of the room setup with a participant **(B)**.

The regulation of droplet and aerosol spread were important considerations to minimize the risk of disease spread. Because participants were engaging in indoor physical activity, bodily secretions like sweat and aerosol accumulation due to heavy breathing were likely to occur. To address this, air filtration, ventilation, and airflow have been enhanced in our laboratory space. The portable air purifier with high efficiency particulate air (HEPA) filtration was cycling during all participant visits and purified the room three times per hour. Additionally, an exterior door next to the participants was open, and a fan was positioned to blow the participants respiratory droplets and bodily secretions out of the door (Figure 1). Dry indoor environments can contribute to the spread of the virus so airflow from the open door was a key factor (Raj et al., 2020). Location was a factor in our ability to achieve this indoor/outdoor laboratory set up. Los Angeles, CA has a dry subtropical climate year-round, more commonly classified as a Mediterranean climate. The temperature is mild-to-hot with little rainfall. This temperature allows for this set up to be accomplished without the complication of inclement weather like snow and rain (Climate Data, 2021).

The following sections describe the protocols used for each visit and assessment type: consent, medical screen, questionnaires, blood draw, brain MRI, cognitive testing, fitness testing, VR system preparation, and VR training visit.

#### COVID-19 Screening Prior to On-Campus Visit

Prior to building entry, participants were required to undergo a forehead temperature check and answer an 8-item screening questionnaire (Table 3). The questionnaire was based on the CDC guidelines to inquire about symptoms commonly associated with COVID-19. Participants with self-reported COVID-19 symptoms or three consecutive temperature readings above 100.4 (Centers for Disease Control Prevention, 2017) were not permitted to enter the building. If granted entry, participants were required to wear a mask throughout the visit and while on campus.

#### Virtual Consenting

The informed consent and Health Insurance Portability and Accountability Act (HIPAA) forms were administered remotely *via* Zoom^™^ video conference. The participant was seated in a private testing room with a laptop, a sanitized pen and paper forms of the informed consent and HIPAA. The researcher was seated in a separate private testing room with a laptop. The researcher communicated with the participant verbally and visually using the screen-share function. The forms were reviewed together on screen. Once the review was complete and all of the participant’s questions were answered, the participant and experimenter signed the paper forms of the informed consent and HIPAA, and a copy was given to the participant.

#### Medical Screening

The screening began with an interview followed by physiological measurements. The medical history interview was 10–15 min and was completed face-to-face in a testing room with six feet between participant and researcher. The researcher wore a surgical mask over their mouth and nose and a face shield at all times to provide an added safeguard when distance of 6 ft could not be achieved. The participant wore a mask over their mouth and nose. After the interview, the researcher measured blood pressure with an automated arm cuff, which was sanitized with 75% ethanol after use. We substituted the manual blood pressure cu with an automatic cu to minimize person-to-person contact and allow the participant to take their own blood pressure with the push of a button.

#### Trial Outcomes Data Collection

Our study protocol included in-person assessments at screening, baseline, and follow-up. These assessments included blood draws, brain MRI scans, cognitive testing, physical testing, and questionnaires. These assessments were adapted to minimize the risk of COVID-19 exposure and transmission.

##### Online Questionnaires

All questionnaires were completed by participants at-home or administered in-person online *via* a handheld tablet to reduce in-person time and exposure. If administered in person, the participant completed the questionnaire alone in a private testing room and the tablet was sanitized with an electronic wipe after use.

##### Blood Draw

The participant’s blood was drawn by a phlebotomist on a sanitized MRI scanner bed. The MRI technologist sanitized the scanning room with 70% ethanol solution and germicidal alcohol wipes after use with a 30-min buffer between participants to allow the ethanol solution to dry.

##### MRI Brain Scan

The participant was cleared of any metal or MRI contraindications and provided with an MRI compatible face mask. The participant was scanned on a sanitized MRI scanner bed, and all fabrics were disinfected by a laundry service after each use. All head coils, cushions, and pads were sanitized with 70% ethanol solution and germicidal alcohol wipes after use. The sanitizing procedure includes disinfecting the scanner bed and cushions by saturating with ethanol solution then allowing 30 min for it to dry. Additionally, scrubbing high contact surfaces in the scanner room, wiping dry with towels/paper towels, and discarding soiled linen in proper compartments for cleaning pick up. Utensils used include a spray bottle, towels, trash bins, linen hampers, and gloves. This disinfecting procedure takes ~15 min. Special attention should be paid to foam cushions that have direct contact with participants faces while lying in the scanner. Attention should also be paid to any crevices on the scanner bed. While cleaning, appropriate PPE should still be worn including surgical face masks over mouth and nose, face shield, and gloves.

##### Cognitive Testing

Prior to COVID-19, a one-hour cognitive test session was conducted between a researcher and participant face to face in a private cognitive testing room. Due to the length of our cognitive test battery, a remote online cognitive testing protocol in which researcher and participant are in separate rooms was created. This was achieved by using online video conferencing application Zoom^™^, remote computer control application TeamViewer^™^, two laptops, and an Elmo 1433 Model OX-1 Visual Presenter^™^ document camera. Prior to the participant entering their private testing room, the room was sanitized with 75% ethanol wipes, and a laptop and document camera were set up. The researcher provided the participant with pens, pencils, labeled cognitive test handouts, hand sanitizer, and alcohol and electronic wipes for their personal use. The procedure to sanitize rooms after use included scrubbing high contact surfaces with alcohol wipes, wiping dry with towels/paper towels, and discarding any disposable used materials. This disinfecting procedure takes ~15 min. Special attention should be paid to non-obvious high-contact surfaces like doorknobs, sink handles, and light switches.

##### Fitness Testing

Physical testing was administered in a large 2,200 sq. ft. open exercise room. Two researchers were present in the room along with the participant. Physical function testing included a stationary bike test and modified physical function tests. During these assessments, researchers wore face shields over their face masks due to the necessary close proximity at certain points during testing. A distance of more than six feet was maintained when safe and possible. Participants wore face masks over their mouth and nose during testing. Each piece of equipment used for physical testing was sanitized with 75% ethanol after use.

#### VR System Preparation

The following procedures were followed during in-lab VR equipment and training sessions to minimize the risk of COVID-19 exposure and transmission.

##### Disinfecting VR Headset

The face cushion on the VR headset was replaced with a polyurethane foam cushion, which can be disinfected more easily than the standard face cushion (VR Cover, 2019). Before and after each use, the polyurethane foam cushions were disinfected with antibacterial cleaning wipes, and the cloth head strap was washed with standard antibacterial soap. Cleaning wipes were also used to disinfect all visible surfaces on the headset, including the headphones, nose guard, lens, and outer shell. As a final step, the headset was placed under medical-grade UV-C light for 60 s using CleanBox^™^ technology. Briefly, CleanBox^™^ is a hygiene technology that utilizes UV-C light to decontaminate commercial VR headsets, with 99.9% efficacy on COVID-19 based on independent laboratory testing. However, the cone of UV-C light on the CleanBox^™^ is only effective over short distances so additional cleaning was conducted to ensure complete sanitization of the headset.

##### Disinfecting Equipment

Before and after each visit all equipment was disinfected. The VR bike seat was cleaned with gym equipment cleaner, and the handlebars and cup holder were cleaned with 75% ethanol wipes. All counters, tables, surfaces, chairs, doorknobs, and light switches were disinfected with 75% ethanol wipes. The chest heart rate monitor strap was washed with antibacterial soap after use. The participant’s designated cubby was cleaned with 75% ethanol wipes before any of their sanitized items were placed back inside of it.

#### VR Training Visit

##### Training Visit Protocol

The participant arrived in the parking lot and notified the research staff of their arrival *via* phone or email. A researcher wore a face mask and met the participant in the parking lot to administer the COVID-19 screening questionnaire. If the participant passed the questionnaire, they were provided with a green sticker with the date indicating that they have passed the COVID-19 screening on that day. The researcher then escorted the participant to the laboratory abiding by COVID-19 hallway safety/capacity regulations to minimize the risk of disease exposure to students, faculty, and staff. Once the participant entered the lab, they were instructed to place their belongings in the designated belonging area. The participant was provided with a designated cubby and instructed to put on their individual heart rate monitor. The study staff measured the participant’s blood pressure using an automated arm cu while in a seated position. The study staff recorded blood pressure and heart rate and then escorted the participant over to the stationary VR bike. The participant sat on the VR bike and placed the VR HMD (with their individual face cushions that were pre-set before their visit begins) on their head. The researcher stood six feet away during the training session. The study staff started the program and the participant cycled on the stationary bike in the VR environment for a 60-min period including breaks between each session. The researcher remained six feet away from the participant throughout their 60-min cycling session. Once the participant finished the session, the study staff asked them two short questionnaires pertaining to their session that day. The participant was instructed to remove the VR HMD and rest it on the bike handlebar, step off the bike, remove their heart rate monitor and place it in their designated cubby. The participant was then escorted out of the building and back to their car by study staff.

## RESULTS

Overall, all five participants were able to participate in the VR intervention while wearing the required PPE. However, two participants reported initial feelings of claustrophobia and general discomfort while wearing a facemask and VR headset simultaneously. These feelings increased with physical exertion, until both participants momentarily pulled the facemask away from the face for breathability. Masks were then placed back over the mouth and nose before resuming the study. Most participants acclimated to this and did not move their mask for breathability after the first few sessions. Other existing safeguards including six feet distance, air filtration, and an outdoor setup help to mitigate the risk of spread. Another alternative to help this problem is to offer different types of masks like n95 and kn95 which sit farther away from the face. Furthermore, all participants briefly removed the facemask to drink water during breaks. Disposable VR eye mask covers were uncomfortable for participants to wear in the presence of face masks. When worn together, both masks would interfere with one another causing either the eye mask to be pushed upward into the participants field of view, or the face mask to be pushed downward exposing more of the face. Although studies have shown that it is safe and feasible for healthy subjects to participate in moderate-strenuous aerobic physical activity with a face mask on (Epstein et al., 2020), there is no information about the feasibility of wearing a face mask in the presence of an eye mask for VR. As an alternative to disposable VR eye mask covers, participants were assigned an individual VR face polyurethane foam cushion for personal use during training. This was made possible with study funding to mitigate the issue of sharing equipment. Participants reported that these cushions were more comfortable than disposable VR eye masks.

None of the five participants or three study staff reported any COVID-19 symptoms on our 8-item checklist or reported a positive COVID-19 test. COVID-19 testing was not mandatory for participants or study staff. Voluntary testing was available to all staff at no cost through university resources. More than 40 COVID-19 tests were completed by the study staff over a 6-month period.

All participants were surveyed about the study safety regulations in response to COVID-19. Five questions were administered using a Likert scale of 1–4. Four of the questions included options 1 indicating not at all, two indicating somewhat/slightly, three indicating very, and four indicating extremely. One question was administered using one as strongly disagree, two as disagree, three as agree, and four as strongly agree. No neutral “no response” option was provided. Four (80%) participants strongly agreed that the required PPE for this VR intervention was comfortable while one (20%) participant strongly disagreed. Two (40%) participants responded that they felt the research staff PPE was extremely important, two (40%) participants responded very important and one (20%) slightly important. Three (60%) participants reported that they were extremely satisfied with the established sanitization and safety protocols for the study while two participants (40%) were very satisfied. All (100%) of the participants reported that the research team addressed their concerns with returning to research “extremely well.” Participants reported to be either “very likely” (40%) or “extremely likely” (60%) to participate in another virtual reality study during the COVID-19 pandemic. Although the 4-point Likert scale provided a limited number of response options, studies report “there is no major difference in terms of means, standard deviations, item-item correlations, item-total correlations, Cronbach’s alpha or factor loadings” between a 4-, 5-, 6-, or 11- point Likert scales (Shing-On, 2011).

There are some notable limitations of this case study of feasibility of VR training during the era of COVID-19. Due to our small sample size of five participants, this approach may not be generalizable to a broader population. Our study sample is composed of women only; therefore, the applicability of the approach may differ for men. Additionally, all healthy older adults were studied, so other considerations may be necessary for clinical populations.

### Real-World Considerations of Our Study Design

Many laboratories are not equipped with an exterior door to increase airflow and reduce disease transmission. In the absence of access to an exterior door, other precautions can be taken, including opening windows or enhanced use of heating, ventilating, and air conditioning (HVAC) systems. HVAC systems help to dilute the concentration of pathogens in a closed room and assist with air change rates (Ascione et al., 2021). Increased distancing of persons may also mitigate some of the concerns of droplet transmission. The use of a fully outdoor research space, such as a quad in a building complex, can also be explored.

Resuming our study in accordance with this new safety protocol required a significant financial investment of ~$3,000 USD. This included the cost of the CleanBox^™^, the HEPA air filter, individual plastic cubbies for participant storage, individual face cushions, face masks, face shields, gloves, head thermometers etc. We consumed cleaning products quickly due to our cleaning protocol, totaling ~$60 per month.

### Real-World Challenges

All five of our participants completed the study in its entirety without significant disruptions. However, participant adherence and study continuity may be impacted by changing city, county, and state regulations regarding essential and non-essential activities during the pandemic. We did encounter some PPE-related challenges during the trial. Participants initially had difficulty wearing their face mask for the duration of the VR cycling experience due to feelings of claustrophobia. These participants would pull their mask away from their face to breathe without obstruction for a few seconds. Our participants acclimated and were able to complete the entire session with their face mask on; however, this may not be possible for all participants or clinical populations. Some of the safeguards included 6 ft distance between the participant and the masked and shielded researcher, an open door and fan to regulate air flow, and a HEPA air filter. Running an in-person study in the era of COVID-19 is a risk in itself, so the overarching challenge was preventing disease spread. Careful and thorough cleaning is necessary in order for this to be successful. Additionally, if a study is time-sensitive but a participant or study staff contracts COVID-19, their progress would have to be delayed at least 10–14 days based on the current CDC guidelines (Centers for Disease Control Prevention, 2019). This protocol could be further improved if weekly COVID-19 tests of participants was conducted but could be a barrier to participation.

## CONCLUSIONS

This study demonstrates the feasibility of multiple in-lab visits with a mid-to-late life older adult cohort. Our research team safely completed more than 200 virtual reality training visits and 17 assessment visits by following the established protocol. During this active data collection time, a California state-wide stay-at-home order was in place. Research was deemed essential work at this time and university regulations allowed the resumption of research with precautions in place. At the start of the study on August 17, 2020 there were 216,683 cumulative cases, 1,520 daily cases, 5,257 cumulative deaths, and 39 daily deaths in Los Angeles county. At the end of the study on December 17, 2020 there were 618,186 cumulative cases, 15,792 daily cases, 8,968 cumulative deaths, and 136 daily deaths due to COVID-19 (LA County COVID-9 Surveillance Dashboard, 2020). During this time period none of our participants reported any known COVID-19 symptoms, and none of our research staff received a positive COVID-19 test result. Two participants expressed discomfort while wearing the facemask. However, their self-reported discomfort of face masks did not prevent participation in the study, enabling full adherence to the safety protocols in place. Abiding by the guidelines put forth by CDC and WHO we demonstrated that in-lab research is feasible with prescribed safety precautions and safeguards. We conclude that continued VR clinical trial research during the COVID-19 pandemic is possible if specific safety protocols are established and followed. The safety and sanitization protocol described in this case series may serve as a guideline for other VR studies launching in the era of COVID-19.

## Figures and Tables

**TABLE 1 |:** Study protocol developed following CDC COVID-19 guidelines.

CDC COVID-19 guidelines	Guidelines addressed
Wash hands	Study staff are required to wash their hands thoroughly with antibacterial soap and water throughout the day. There are two sink hand washing stations immediately outside of the laboratory and three hand sanitizing stations inside of the laboratory.
Avoid close contact	Study staff and participants are required to maintain a 6 ft distance. Room occupancy is reduced to a maximum of three people in our laboratory space at a given time.
Cover mouth and nose with a mask when around others	Research team members are required to wear surgical masks covering their mouth and nose at all times and face shields when participants are in the laboratory. All participants are required to wear a face covering over their mouth and nose while in any USC facility.
Cover coughs and sneezes	Signage has been placed throughout all USC buildings to promote proper cough/sneeze etiquette. Study staff and participants are encouraged to follow these recommendations.
Clean and disinfect equipment and surfaces	The laboratory is cleaned and disinfected with 75% alcohol wipes and bleach germicidal wipes before, after, and between subject visits each day based on CDC guidelines. This includes high touch surfaces, such as doorknobs, counters, tables, and chairs. Each participant is provided a personal VR HMD cushion set, which is stored in a designated cubby. Cubbies and cushions are sanitized with 75% alcohol after use. The VR bike handlebars, cup holder, and seat are sanitized with 75% ethanol wipes after use. Each participant is provided with a heart rate monitor strap, which is washed with antibacterial soap and stored in the designated cubby. The blood pressure cuff is sanitized with 75% ethanol after each use. The “Cleanbox^™^” is a recently developed UVC light decontamination device for VR HMDs and is used to disinfect the small cracks and crevices in the VR headset. Published data describes 99.9% effectiveness of UVC light (Casini et al., 2019; Otter et al., 2020).
Monitor your health daily: Trojan Check and COVID-19 tests for study staff	Study staff complete an online health screening before entering any campus building. If passed, a QR building access code is provided to gain entry. A similar health screening questionnaire is administered to study participants prior to building entry. If passed, a green dated COVID-19 screening sticker is provided and worn on the outermost layer of clothing. If study staff or participants display symptoms, they are required to stay home until they receive a negative test result, and all symptoms cease. If one tests positive for COVID-19, they are required to quarantine for 14 days and have three negative test results before they may return to campus. That staff member is obligated to notify the principal investigator of the study to proceed with COVID-19 contact tracing. The research staff undergoes frequent COVID-19 testing offered for free to faculty, staff and students at several campus locations.

**TABLE 2 |:** Study protocol developed following WHO COVID-19 guidelines.

WHO COVID-19 guidelines	Guidelines addressed
Make sure your workplaces are clean and hygienic. Surfaces and objects need to be wiped with disinfectants regularly.	Our research staff sanitizes all surfaces and equipment with 75% alcohol wipes between each participant.
Promote regular and thorough handwashing. Place sanitizing hand rub dispensers in prominent places around the workplace. Make sure these dispensers are regularly refilled.	Hand sanitizing dispensers have been placed in 3 locations throughout the laboratory. These dispensers are regularly filled.
Display posters promoting handwashing.	CDC posters with COVID-19 hand-washing guidelines are posted throughout all campus buildings.
Make sure that staff, contractors, and customers have access to places where they can wash their hands with soap and water.	There are two sinks that are accessible to participants and staff outside of the laboratory on either side. The sink knobs are disinfected between participants.
Promote good respiratory hygiene in the workplace. Display posters promoting respiratory hygiene. Combine this with other communication measures such as briefing at meetings, and information on the intranet.	The University provides additional guidance *via* email weekly regarding COVID-19 safety measures and on-campus operation. Respiratory hygiene posters are displayed throughout all university buildings. Research staff is encouraged to follow these recommendations.
Ensure that face masks or paper tissues are available at your workplaces, for those who develop a runny nose or cough at work, along with closed bins for hygienically disposing of them.	Disposable surgical face masks are available for participants and staff use each day. We have paper towels/tissues available at all times and biohazard trash bins with lids for disposing of any materials containing participant bodily fluids/secretions.
Advise employees and contractors to consult national travel advice before going on business trips.	All business travel is suspended, all international travel is restricted, and all domestic travel is discouraged unless essential.
Promote the message that people need to stay at home even if they have only mild symptoms of COVID-19.	Staff members are required to complete a health screening each day and are required to stay home if they are displaying even mild symptoms.
Display posters with this message in your workplaces. Combine this with other communication channels commonly used in your organization or business.	Posters regarding symptoms are displayed throughout university buildings encouraging faculty and staff to stay home if they are sick.

**TABLE 3 |:** Eight-item COVID-19 participant screening questionnaire.

Eight-item COVID-19 screening questionnaire
In the past 14 days have you had close contact with a person known to have the novel coronavirus?
In the past 14 days have you, yourself, had the novel coronavirus?
**Do you have ANY of the following symptoms:**
Fever of 100° or higher?
Muscle soreness or headaches accompanied by shaking, chills, and fever?
New onset of upper respiratory symptoms, running nose, sore throat along with fever?
New onset of gastrointestinal symptoms such as abdominal pain, diarrhea, or vomiting with fever?
Loss of taste or smell?
Conjunctivitis with a fever?

Eight-item questionnaire administered to research participants before each research visit and authorization to enter any campus building. If participants respond “Yes” any of the questions, they are not allowed to engage in research or enter campus facilities.

## Data Availability

The raw data supporting the conclusions of this article will be made available by the authors, without undue reservation.
